# Nurses’ and midwives’ knowledge, attitudes, and acceptance regarding human papillomavirus vaccination in Ghana: a cross-sectional study

**DOI:** 10.1186/s12912-020-00530-x

**Published:** 2021-01-06

**Authors:** Nancy Innocentia Ebu, Gifty Esinam Abotsi-Foli, Doreen Faakonam Gakpo

**Affiliations:** 1 Department of Adult Health, School of Nursing and Midwifery, University of Cape Coast, Cape Coast, Ghana; 2 Department of Obstetrics and Gynaecology, Korle-Bu Teaching Hospital, Accra, Ghana; 3 Kibi Government Hospital, Kibi, Ghana

**Keywords:** Attitudes, Cervical Cancer, Ghana, HPV vaccination, Knowledge, Nurses and midwives

## Abstract

**Background:**

Nurses and midwives play important roles in educating the public on cervical cancer prevention strategies.

**Aim:**

This study sought to assess nurses’ and midwives’ knowledge of, attitudes towards, and acceptance of human papillomavirus (HPV) vaccination in relation to their background characteristics.

**Methods:**

A descriptive cross-sectional study using questionnaires was conducted with a convenience sample of 318 female nurses and midwives, ages 20 to 59, at the Korle-Bu Teaching Hospital in Ghana. The data were summarised using frequencies, percentages, chi-square tests, and Fisher’s exact tests.

**Results:**

The results indicated that 41.5% (*n* = 132) of the participants had high levels of knowledge about cervical cancer risk factors, and 17.6% (*n* = 56) of the respondents had received at least one dose of the HPV vaccine. Reasons for receiving the HPV vaccination included advice from a colleague (12.9%, *n* = 41) and perceived threat of cervical cancer (11.7%, *n* = 37). Of the 262 respondents who had not been vaccinated, 24.45% (*n* = 78) strongly agreed and 28.0% (*n* = 89) agreed with the statement that there was limited information on HPV vaccination. Also, there were statistically significant associations between age (X^2^ = 23.746, *p* = 0.001), marital status (X^2^ = 14.758, *p* = 0.005), completed level of education (X^2^ = 21.692, *p* = 0.001), and duration of working at the hospital (X^2^ = 8.424, *p* = 0.038) and acceptance of HPV vaccination.

**Conclusions:**

This study demonstrated gaps in knowledge about cervical cancer risk factors and attitudes towards HPV vaccination, indicating the need for targeted measures to improve knowledge and attitudes. Also, measures to increase acceptance of HPV vaccination among nurses and midwives should consider their sociodemographic characteristics.

## Introduction

HPV infections are most common in young adults, but it generally takes decades for high-risk HPV infections to develop into cervical cancer. HPV is a sexually transmitted infection and the causative agent of cervical cancer [1, 2]. It is estimated that HPV causes 100% of cancer of the cervix [3], 75.8% of anal cancer, 73.3% of vaginal cancer, 40.5% of vulvar cancer and 49.1% of penile cancer [4]. It is well documented that HPV types 16 and 18 may cause more than 70% of all cervical cancer globally [3]. In Ghana each year, 3151 women are diagnosed with cervical cancer, and 2119 die from the disease [5]. Based on estimates from the subregions, the prevalence of HPV infection, mostly types 16 and 18, among women with normal cytology is 4.3%, low-grade cervical lesions are found in 24.3% of women, and high-grade cervical lesions are diagnosed in 35.6% [5]. However, a study conducted in the Korle-Bu Teaching Hospital (KBTH) with 256 confirmed cases of cervical cancer reported a high prevalence of 47.4% for HPV type 18, 42.2% for type 59 and 37.4% for type 45 [6].

Primary and secondary prevention strategies, including HPV vaccination, screening, and early treatment of precancerous lesions, are critical to decrease incidence and mortality. High-income countries have been successful in decreasing the morbidity and mortality of cervical cancer by 70% through effective cervical cancer screening interventions [7]. Nonetheless, cervical cancers are not diagnosed early in low- and middle-income countries due to a lack of information about the disease and inadequate vaccination and screening programmes [8, 9].

HPV vaccination is one of the preventive strategies for cervical cancer. Although some high-income countries have adopted two novel prophylactic vaccines and other secondary preventive methods [10] to deal with the situation, these interventions may not be readily available in low-resource settings due to limited infrastructure for health care delivery [11]. The World Health Organisation (WHO) has recommended that countries integrate HPV vaccination into their routine vaccination programmes [7]. HPV vaccination is recommended for adolescents 11 to 13 years, and it could be given as early as age 9. In the United States, catch-up vaccination is recommended for females through age 26 and for some special populations [12, 13].

The HPV Bivalent Vaccine Cervarix, manufactured by GlaxoSmithKline, can be administered in two to three doses based on the age of the person [12]. In 2013, Ghana’s government, with support from the Global Alliance for Vaccines and Immunisations (GAVI), introduced the HPV vaccination, which was administered to girls ages 9 to 11 in selected districts [14]. Following this initial vaccination, there has not been any structured programme for HPV vaccination at the national level. However, there are a few public and private health facilities that provide HPV vaccination, which is normally administered by either trained nurses or midwives.

Nurses’ and midwives’ knowledge of HPV vaccination is critical, as these professionals play essential roles in disease prevention and health promotion by educating the public through culturally-relevant health education and promotion strategies. A previous study involving nursing, medical, and dental students reported that participants with at least a moderate or high knowledge of cervical cancer had a greater chance of intending to receive HPV vaccination. Additionally, a positive attitude about HPV was associated with intention to screen [15]. It is plausible to assume that when nurses and midwives are well informed, they may have positive perceptions about HPV vaccination. A systematic review on the acceptability of HPV vaccines in sub-Saharan Africa concluded that endorsement of the vaccine by health professionals will improve its acceptance [16]. Therefore, high-quality recommendations by healthcare providers may influence HPV vaccination behaviour [17]. For nurses and midwives to effectively make those recommendations, a board of experts recommended training healthcare providers to have high expectations and develop appropriate culturally-relevant materials on HPV vaccination [18]. However, several barriers could hinder nurses and midwives from participating in HPV vaccination. These barriers include gaps in knowledge of HPV vaccination [19]. Despite positive attitudes, inadequate knowledge about HPV vaccination and testing could lead to misinformation and possible stigma since health professionals constantly interact with the public [20]. A longitudinal study conducted in Turkey reported that the reluctance of nurses to obtain HPV vaccination was linked with the cost and distrust about the efficacy and safety of the vaccine [21].

Despite these factors that could impede HPV vaccination among health professionals, nurses and midwives are viewed as role models in matters relating to health [22], including the practice of health-enhancing behaviours. A systematic review demonstrated a high level of acceptability of the HPV vaccine in the sub-Saharan African region; however, it highlighted significant gaps in knowledge and awareness that require intensification of public education on cervical cancer, HPV and HPV vaccines [23]. Furthermore, there has been a recommendation for more public education to enable the public to understand the synergy between HPV and cervical cancer [24]. Although nurses and midwives are important stakeholders in HPV prevention, little is known about nurses’ and midwives’ knowledge, attitudes, and acceptance of HPV vaccination. In Ghana, previous studies on cervical cancer prevention have examined the knowledge and health beliefs of women regarding cervical cancer and cervical cancer screening [8, 25], the prevalence of HPV [6] and the epidemiology of cervical HPV infection among women [26]. The level of knowledge of nurses and midwives on cervical cancer risk factors and the acceptance of HPV vaccination is unclear. Given the role nurses and midwives play in disease prevention, it is assumed that when they are knowledgeable about the disease, they are able to take appropriate action and counsel their clients and the general public with cultural sensitivity about appropriate health actions. Therefore, this study sought to 1) assess the level of knowledge of nurses and midwives on cervical cancer risk factors, 2) determine the proportion of nurses and midwives who had at least one cervical cancer screening, 3) determine the proportion of nurses and midwives who had received at least one dose of HPV vaccination, 4) identify their reasons for receiving HPV vaccination, and 5) identify their barriers to HPV vaccination. This study also explored the association between sociodemographic factors and acceptance of HPV vaccination by nurses and midwives at the KBTH.

## Methods

### Study design and setting

A descriptive cross-sectional study was conducted at the KBTH. The KBTH is a premier health facility located in the southern part of Ghana. It is the third-largest hospital in Africa and a major referral centre in Ghana. The facility has a bed capacity of approximately 2000 and 17 clinical and diagnostic departments. It also provides sophisticated and scientific investigative procedures and specialisation in various fields, such as neurosurgery, dentistry, eye/ear/nose and throat (ENT), renal surgery, orthopaedics, oncology, dermatology, cardiothoracic surgery, radiotherapy, paediatric surgery, reconstructive plastic surgery and burns. It has an average daily attendance of 1500 clients and 250 admissions. The choice of this teaching hospital was appropriate because it is a major referral point in the country and has a significant number of female nurses and midwives.

### Population

The target population of this study consisted of female nurses and midwives at KBTH. Data from the KBTH records unit indicate that there are 1750 female nurses and midwives on staff [27]. This study included female nurses and midwives ages 20 to 59 employed by KBTH, and possessing at least a State Registered Nursing certificate or a diploma in either nursing or midwifery. Those with a certificate in community health nursing, nursing assistants, and males were excluded from the study. Although ACIP left the decision to vaccinate persons ages 27 to 45 in the hands of clinicians [13], this study included nurses and midwives up to 59 years of age. The focus of the study was on the knowledge of nurses and midwives about the risk factors for cervical cancer and whether they had received at least one dose of the HPV vaccine. We believe that positive attitudes of the sampled population towards HPV vaccination may have a positive impact on the health education and recommendations they provide to women.

### Sample and sampling procedure

The sample size required for the study was determined using Yamane’s 1998 formula for sample size determination [28].

$$ \mathrm{n}=\frac{N}{1+N\;\left(\infty \right)}\;2 $$

Where:

n = sample size

N= study population

α= margin of error, which is 0.05 with a significance level of 95%.

Based on this formula, a sample size of 325 was anticipated for the study. We added 10% to cover any uncertainties [29]. A total of 357 nurses and midwives were expected to participate in the study. The simple random sampling procedure with the replacement method was used to select 10 wards/units out of 16 major wards/units. These are the surgical wards, medical wards, surgical medical emergency, intensive care, maternity floors, obstetric recovery, gynaecological theatre, gynaecological OPD, and gynaecological wards and maternity OPD. Although most of these units/wards do not offer cervical cancer screening and HPV vaccination, the nurses do rotate among the different units annually and often interact with sexually active girls and women whom they could counsel about cervical cancer and HPV vaccination. The midwives, however, rotate only in the obstetrics and gynaecology wards. Hence, the nurses and midwives in all these units were included in the study. Based on the number of nurses and midwives in each unit and the sample size for the study, quotas were assigned to each unit. A convenience sampling method was used to collect the data by involving nurses and midwives who were available at the time of data collection and were willing to participate in the study until the quotas for all the units were reached. Although convenience sampling has been used in most hospital-based studies, it could affect the generalisability of the findings [29].

### Study instrument

A questionnaire was used as the data collection tool with items adapted from previous studies [8, 25]. These previous questionnaires were used to determine the knowledge of cervical cancer and cervical cancer screening behaviour of women in Ghana. The literature was reviewed, and some additional questions were added to the survey. The current instrument consisted of the following: knowledge of cervical cancer risk factors, cervical cancer screening, acceptance of HPV vaccination, reasons for receiving HPV vaccination, barriers to HPV vaccination, and the participants’ sociodemographics. The 10 knowledge of cervical cancer risk factors were multiple sexual partners, HPV infection, unprotected sexual activity, genital herpes, smoking, age, race, ethnicity, oral contraceptives, and exposure to diethylstibestrol. The responses to these items were Yes, No and Don’t Know. Regarding the cervical cancer screening test, the question asked was, “Have you had at least one Pap smear test?” Participants were required to respond either Yes or No. The acceptance domain was measured by a single item, “Have you received at least one dose of HPV vaccination?”, which elicited either Yes or No response.

The five reasons for accepting the HPV vaccination were as follows: advice from family or friends, advice from a colleague health worker, perceived threat of cervical cancer, media discussion on cervical cancer, and a friend or colleague who died from the disease. The items in this domain were on a four-point Likert scale: strongly agree, agree, disagree, and strongly disagree. This elicited multiple responses from the participants.

The barriers to HPV vaccination domain consisted of 12 items: the vaccine is expensive, going for the HPV vaccine is a waste of time, the unavailability of vaccination sites, there is limited information on the HPV vaccine, I don’t know what the HPV vaccine is all about, there are no health education programmes to promote HPV vaccination, HPV vaccination is not necessary, I do not have money to take the vaccine, fear of adverse effects of the vaccine, risk of becoming infected with HPV, anticipation of family disapproval, and fear of experiencing pain during injections. These barrier items were rated on a four-point Likert scale: strongly agree, agree, disagree, and strongly disagree.

The research instrument also covered respondents’ sociodemographic characteristics, including age, marital status, religion, completed level of education, and duration of working in the hospital.

### Validity and reliability

The instrument was pretested on 50 nurses and midwives in a nearby health facility. Ambiguities with some of the items were detected, and questions were reworded. For example, “Have you ever had cervical cancer vaccination”? was changed to “Have you received at least one dose of HPV vaccination”? to improve clarity. The instrument was shown to at least two experts to determine the adequacy of the items in measuring the intended concepts. Reliability for the items on the knowledge domain was assessed using Kuder-Richardson Formula 20. The coefficients of reliability for the Likert-type items were determined using Cronbach’s alpha. The following reliabilities were obtained for the various domains: knowledge = .806, reasons for accepting HPV vaccination = .711, and barriers = .824.

### Data collection procedure

Three registered nurses were trained to assist with the data collection. The data collection took approximately six weeks from September to October 2018. The questionnaires were administered to the participants after the purpose of the study had been explained to them and they had voluntarily agreed to participate in the study. Some of the participants completed the questionnaires in the nurses’ lounge after they had finished their shifts. Most participants completed their questionnaires off-site and returned them to the research assistants on their next shifts.

### Data analysis

Data were analysed using frequencies, percentages, chi-square and Fisher’s exact tests. Respondents with knowledge scores of 59% and below were categorised as having low knowledge about cervical cancer risk factors. Those with a score of 60 to 79% were considered to have moderate knowledge, while those with a knowledge score of 80% and above were considered to have high knowledge. These cut points were based on a previous study [30]. With those scores in place, the level of knowledge of participants on cervical cancer risk factors was summarised using frequency counts and percentages. The proportion of the participants who had at least one Pap smear test and at least one dose of HPV vaccination, the reasons for receiving HPV vaccination, and the barriers to HPV vaccination were summarised using frequency counts and percentages. Furthermore, the associations between sociodemographic factors and acceptance of HPV vaccination were assessed using chi-square and Fisher’s exact tests.

## Results

The results in Table 1 indicate that less than half of the respondents (47.5%, *n* = 151) were aged 20 to 29 years. In relation to their marital status, 45.0% (*n* = 143) were married, while 43.7% (*n* = 139) had never married. In regard to religion, 82.7% (*n* = 263) were Christians, while 15.4% (*n* = 49) were Muslims. In connection with respondents’ level of education, 48.1% (*n* = 153) had a 3-year diploma, while 37.1% (*n* = 118) had a bachelor’s degree. The results further indicate that 37.1% (n = 118) had been working in the teaching hospital for one to five years, while 10.7% (*n* = 34) had been working in the facility for over 11 years.

**Table 1:** Sociodemographic characteristics of the respondents *N* = 318

Variables	Frequency	Percentage
**Age range**		
20–29	151	47.5
30–39	118	37.1
40–49	29	9.1
50–59	20	6.3
**Marital status**		
Married	143	45.0
Never married	139	43.7
Separated	15	4.7
Divorced	12	3.8
Widowed	9	2.8
**Religion**		
Christians	263	82.7
Muslims	49	15.4
African traditional	6	1.9
**Level of education**		
Masters	16	5.0
Bachelors	118	37.1
3-year Diploma	153	48.1
Certificate	31	9.7
**Work experience**		
Less than a year	92	28.9
1–5 years	118	37.1
6–10 years	74	23.3
Over 11 years	34	10.7

Regarding knowledge of cervical cancer risk factors, 41.5% (*n* = 132) of the participants had high knowledge of the risk factors for cervical cancer, while 29.6% (*n* = 94) had low knowledge, as shown in Table 2. Most of the respondents (55.3%, *n* = 176) indicated that they had at least one Pap smear test, while 44.7% (*n* = 142) had not had a test. Additionally, only 17.6% (*n* = 56) had received at least one dose of HPV vaccination, while 82.4% (*n* = 262) had not received a dose.

**Table 2:** Level of knowledge of female nurses and midwives on cervical cancer risk factors N = 318

Knowledge	Frequency	Percentage
High	132	41.5
Moderate	92	28.9
Low	94	29.6
Total	318	100.0

Table 3 shows that 6.3% (*n* = 20) strongly agreed and 6.6% (*n* = 21) agreed with the statement that they received HPV vaccination because a health worker colleague recommended it. In addition, 6.0% (*n* = 19) strongly agreed and 5.7% (*n* = 18) agreed that they had the vaccination due to the perceived threat of cervical cancer.

**Table 3:** Reasons for receiving HPV vaccination (*n* = 56)

Reasons	Strongly Agree	Agree	Disagree	Strongly Disagree
	f (%)	f (%)	f (%)	f (%)
Advice from family or friends	14 (4.4)	13 (4.1)	26 (8.2)	3 (0.9)
Advice from colleague health worker	20 (6.3)	21 (6.6)	14 (4.4)	1 (0.3)
Perceived threat of cervical cancer	19 (6.0)	18 (5.7)	13 (4.1)	6 (1.9)
Media discussion on cervical cancer	10 (3.1)	15 (4.7)	20 (6.3)	11 (3.5)
A friend or colleague died from it	9 (2.8)	14 (4.4)	17 (5.3)	16 (5.0)

In connection with the barriers to obtaining HPV vaccination, Table 4 shows that 24.45% (*n* = 78) strongly agreed and 28.0% (*n* = 89) agreed with the statement that there was limited information about HPV vaccination. Additionally, 21.7% (*n* = 69) strongly agreed and 23.9% (*n* = 76) agreed with the statement that the vaccine was expensive. Additionally, 10.1% (*n* = 32) and 22.3% (*n* = 71) strongly agreed and agreed, respectively, with the statement that fear of experiencing pain during injection was a barrier to seeking HPV vaccination. Fear of adverse effects of the vaccination was also cited, as 15.1% (*n* = 48) strongly agreed and 20.8% (*n* = 66) agreed to that assertion. However, 35.2% (*n* = 112) disagreed and 31.8% (*n* = 101) strongly disagreed with the assertion that anticipation of family disapproval was a barrier to HPV vaccination.

**Table 4:** Barriers to obtaining HPV vaccination by female nurses and midwives (n = 262)

Variables	Strongly Agree	Agree	Disagree	Strongly Disagree
	f (%)	f (%)	f (%)	f (%)
The vaccine is expensive	69 (21.7)	76 (23.9)	84 (26.4)	33 (10.4)
Going for HPV vaccination is a waste of time	12 (3.8)	29 (9.1)	102 (32.1)	119 (37.4)
Unavailability of vaccination sites	53 (16.7)	72 (22.6)	81 (25.5)	56 (17.6)
There is limited information on HPV vaccine	78 (24.5)	89 (28.0)	64 (20.1)	31 (9.7)
I do not know what the vaccination is all about	23 (7.2)	56 (17.6)	112 (35.2)	71 (22.3)
There are no health education programmes to promote HPV vaccination	68 (21.4)	83 (26.1)	65 (20.4)	46 (14.5)
HPV vaccination is not necessary	16 (5.0)	28 (8.8)	100 (31.4)	118 (37.1)
I didn’t have money to take the vaccine	41 (12.9)	54 (17.0)	102 (32.1)	65 (20.4)
Fear of adverse effects of the vaccine	48 (15.1)	66 (20.8)	96 (30.2)	52 (16.4)
Risk of becoming infected with HPV	33 (10.4)	43 (13.5)	112 (38.4)	64 (20.1)
Anticipation of family disapproval	13 (4.1)	36 (11.3)	112 (35.2)	101 (31.8)
Fear of experiencing pain during injections	32 (10.1)	71 (22.3)	87 (27.4)	72 (22.6)

Table 5 shows the associations between sociodemographic factors and acceptance of HPV vaccination. There was a statistically significant association between age (X^2^ = 23.746, *p* = 0.001), marital status (X^2^ = 14.758, *p* = 0.005), completed level of education (X^2^ = 21.692, *p* = 0.001), and duration of working at the hospital (X^2^ = 8.424, *p* = 0.038) in regard to acceptance of HPV vaccination. However, there was no statistically significant association between religion (X^2^ = 1.427, *p* = 0.490) and acceptance of HPV vaccination.

**Table 5:** Associations between participants’ sociodemographic characteristics and acceptance of HPV vaccination *N*= 318

Sociodemographic characteristics	Acceptance of HPV Vaccination			
	No f (%)	Yes f (%)	Chi-square (X^2^)	*P*-Value
**Age**				
20–29	134 (51.1)	17 (30.4)		
30–39	96 (36.6)	22 (39.3)		
40–49	23 (8.8)	6 (10.7)		
50–59	9 (3.4)	11 (19.6)	23.746	0.001
**Marital status**				
Single/Never married	120 (45.8)	19 (33.9)		
Married	119 (45.4)	24 (42.9)		
Divorced	10 (3.8)	2 (3.6)		
Separated	8 (3.1)	7 (12.5)		
Widowed	5 (1.9)	4 (7.1)	14.758	0.005
**Religion**				
Christianity	219 (83.6)	44 (78.6)		
Islam	39 (14.9)	10 (17.9)		
African Traditional	4 (1.5)	2 (3.6)	1.427	0.490
**Completed level of education**				
* NMTC (certificate)	28 (10.7)	3 (5.4)		
*NMTC (diploma)	134 (51.1)	19 (33.9)		
Bachelors	93 (35.5)	25 (44.6)		
Masters	7 (2.7)	9 (16.1)	21.692	0.001
**Duration of working at Korle-bu**				
Less than 1 year	81 (30.9)	11 (19.6)		
1–5 years	101 (38.5)	17 (30.4)		
6–10 years	56 (21.4)	18 (32.1)		
11 years and above	24 (9.2)	10 (17.9)	8.424	0.038

* Significant at the 0.05 significance level *Nursing and Midwifery Training College

## Discussion

The purpose of this study was to assess nurses’ and midwives’ knowledge of, attitudes towards, and acceptance of HPV vaccination in relation to their background characteristics. HPV vaccination is critical in the prevention of cervical cancer. Nurses and midwives play pivotal roles in health promotion and the prevention of diseases. Consequently, their level of knowledge and acceptance of HPV vaccination may have a direct impact on their own and their clients’ health promotion activities. The findings of this study indicate that less than half of the sample studied had a high level of knowledge about cervical cancer risk factors. A possible explanation could be that these health professionals may not have had a continuous professional development programme on cervical cancer prevention. It could also be assumed that their training curriculum did not adequately cover cervical cancer. The present finding is consistent with the findings of a cross-sectional study published in 2014; the study was conducted among midwives, students and patients in Serbia, and it found that their knowledge level was unsatisfactory [31]. Despite the differences in the setting and population characteristics, similarities in the design could have accounted for our similar findings. A high level of knowledge among nurses and midwives about cervical cancer risk factors may facilitate effective health promotion and prevention of cervical cancer. An institution-based cross-sectional study conducted in Ethiopia in 2015 reported that over 80% of health workers knew about cervical cancer disease [32]. Health education on the disease may be widespread among the Ethiopian population.

The findings further showed that approximately half of the sample had at least one Pap smear. This is encouraging, as nurses and midwives who have had the test may be able to provide detailed information about it. Earlier studies conducted in Canada, Nigeria and Kenya affirmed the willingness of women to obtain a Pap smear, and the majority had done so [33–35]. These settings may have well-structured cervical cancer screening programmes. A previous study conducted among women in Elmina, the southern part of Ghana, reported a Pap smear screening rate of only 0.8% [8]. Pap smears are an essential preventive strategy for cervical cancer.

Despite evidence that knowledge about cervical cancer could translate into prevention and screening [36], the acceptance of HPV vaccination by nurses and midwives in the current study was found to be low. Hesitancy on the part of some nurses and midwives sampled in this study might explain the observed finding. Earlier findings from the United States suggested that providers had a perception of hesitancy, which deterred them from regularly recommending HPV vaccination [37]. Additionally, since its introduction in 2013 in Ghana, HPV vaccination has not been readily available in most health facilities in the country, with access limited to a few health facilities in urban areas and no access in rural and under-served communities. Nurses and midwives need a high level of knowledge about HPV vaccination to enable them to make an informed decision regarding acceptance of vaccination. Improving the knowledge of nurses and midwives may increase HPV vaccination rates [37]. In the current study, some of the midwives and nurses who had at least one dose of the HPV vaccine had obtained the vaccine because a colleague advised them to. The perceived threat of cervical cancer was also cited as a reason for receiving HPV vaccination.

Several studies have reported low rates of HPV vaccination [38–40]. In a study conducted in Hong Kong among undergraduate students, it was found that only 13.3% of the students had received HPV vaccination [38]. In Turkey, it was reported that only a few nurses received the vaccine [39]. Although these studies were conducted in different settings, the findings strongly suggest that the problem of low vaccination rates against HPV infection cuts across diverse cultures. Studies conducted in Istanbul, Turkey and South India [39–41] found targeted health education among health professionals positively influenced intention to obtain the vaccine and possibly recommend it to others in the future [40]. Earlier work reported that school nurses emphasised intensive education about HPV, as they were responsible for providing education about HPV vaccination to school children and the general public [42]. Contrary to the low HPV vaccination rates, a systematic review conducted in sub-Saharan Africa reported strong evidence for the acceptability of the vaccine in the region but emphasised extensive gaps in knowledge concerning HPV and cervical cancer [16]. For these knowledge gaps to improve, nurses and midwives have major roles to play.

Furthermore, although nurses and midwives are mandated by their codes of practice to perform their tasks without letting personal values interfere, it seems difficult if not impossible to detach some of these personal values from practice. Given the role of nurses and midwives in ensuring professionalism in dealing with clients to promote safe, effective and client-centred care outcomes [43], these professionals can engage in counselling and health education about HPV vaccination to help clients make informed decisions while not imposing personal values on them.

The findings further suggest that some factors could potentially hinder nurses and midwives from participating in HPV vaccination, including limited information and lack of health education programmes to promote HPV vaccination. An earlier work cited knowledge gaps on the part of healthcare providers as a critical barrier in HPV vaccination, as it affects their ability to effectively engage in patient education [44]. In Ghana, health education on HPV and vaccination is not common in health facilities or in the mass media. Moreover, HPV vaccination has not been incorporated into the school health programme to sensitise adolescents and their parents to it. Health education is critical in building capacity, changing beliefs and allowing individuals to make informed decisions.

Furthermore, 45.6% of the participants viewed the cost of HPV vaccination as expensive. In Ghana, it costs GHC 250 the equivalent of approximately $50 (US) to obtain a dose of HPV vaccination, Cervarix, at the KBTH. Therefore, the three doses of Cervarix vaccine recommended will cost approximately $150 (US). The scope of the National Health Insurance Scheme does not cover HPV vaccination, which could discourage potential participants from obtaining the vaccine, especially if they cannot afford the out-of-pocket costs. Other empirical works have reported cost as a major barrier to accessing HPV vaccination [21, 44]. Efforts to reduce financial barriers may increase the uptake of HPV vaccination. Additionally, given the HPV genotypes prevalent in Ghana [6], there is the need for a vaccine that could prevent the varied types of HPV in Ghana, as Cervarix is effective only against HPV types 16 and 18.

Fear of adverse effects of the vaccination was cited as a barrier by 35.9% of the nurses and midwives. It is worth mentioning that a previous study found nurses’ unwillingness to accept the vaccination to be associated with beliefs about the efficacy and safety of the vaccine [21]. This highlights the need for a comprehensive health education programme about the HPV vaccine among healthcare providers to equip them with essential information that will enable them to make informed choices regarding HPV vaccination. The WHO has emphasised that efforts to address the adverse effects of HPV vaccination require coordinated efforts of stakeholders, including healthcare providers [45].

Also, some of the participants cited fear of experiencing pain during injections as a barrier to HPV vaccination. However, surprisingly, a survey conducted in North Carolina among adolescents showed that half of the sampled population complained of pain after receiving an injection, with 2% of the parents complaining their daughters experienced severe pain after the injection. The parents also reported that the pain their daughters experienced after receiving HPV vaccination was either comparable or less intense than the pain from other vaccines [43]. This finding calls for a sensitisation programme for healthcare providers about the vaccine to clarify negative perceptions some may have regarding the HPV vaccine.

The study also explored the sociodemographic factors associated with the acceptance of HPV vaccination. Age was found to be associated with HPV vaccination. Earlier works found young girls to have favourable attitudes towards HPV vaccination [38, 46]. Young girls can be exposed to HPV infection during their first sexual debut. Therefore, receiving the vaccine, especially before becoming sexually active, may be an important step in preventing HPV infection [47]. The differences in the population characteristics between the present study and earlier studies might have contributed to the observed findings.

In this study, level of education was statistically significantly associated with the acceptance of HPV vaccination. It is known that disparities exist in cervical cancer prevention strategies among the general population, especially across different levels of education [46]. Maternal education is an important determinant of childhood immunisation after other personal factors have been controlled for [48]. Women with lower levels of education are less likely to use health services [49]. It seems that some form of education is necessary to enable women to understand and appreciate some critical health concerns.

In the same way, the duration of working in the hospital was associated with the acceptance of HPV vaccination. A possible explanation could be that those who had more work experience may have had more access to continuing education or professional development programmes on HPV vaccination, learned about HPV and cervical cancer on the job or nursed a patient with that condition. In a study conducted in the United Kingdom, experience and trust in doctors influenced vaccine acceptance [50].

Generally, religion seems to have a great influence on decisions regarding appropriate health behaviour [51]. For example, parents who view religion as essential have been found to have a positive attitude towards HPV vaccination for their daughters in a Buddhist society [52]. Additionally, a web-based survey reported that parents of the Catholic faith had higher chances of vaccinating their daughters compared to parents of other religious faiths [53]. In the present study, religion was not associated with the acceptance of HPV vaccination. A possible explanation could be that the majority of the respondents were Christians, so there was not much variability in the data. Nonetheless, women who regularly attend religious programmes are more likely to use cervical cancer screening services [54].

## Limitations of the study

The respondents were sampled by convenience, so there is the possibility of selection bias. Therefore, the results should be interpreted with caution. This sampling approach, however, was the best method, considering the nature of the study. Although nurses and midwives routinely give other vaccines, this study only focused on HPV vaccination. Therefore, the association between other vaccines and HPV vaccination was not assessed.

## Conclusions

This study demonstrated gaps in knowledge of cervical cancer risk factors and attitudes towards HPV vaccination, which requires targeted measures to improve knowledge and attitudes. Despite the low rates of acceptance of HPV vaccination among the sample studied, perceived threats of cervical cancer and recommendation of HPV vaccination by colleague nurses and midwives motivated some of the participants to receive the HPV vaccination. Moreover, the study identified critical barriers that hindered participants from seeking HPV vaccination, including fear of adverse effects of HPV vaccination, fear of experiencing pain during injections and cost of the vaccine. The provision of a comprehensive training programme on HPV vaccination and safety net intervention to cover the cost of the vaccine could reduce barriers and increase uptake. This study also highlighted the need for stakeholders to consider important sociodemographic factors in efforts to increase the acceptability of HPV vaccination.

## Data Availability

The dataset upon which the findings and conclusions of this study are based can be obtained from the corresponding author under reasonable request.

## Abbreviations

HPV: Human Papilloma Virus
KBTH: Korle-Bu Teaching Hospital
Pap: Papanicolaou
